# Utilizing EEG to Explore Design Fixation during Creative Idea Generation

**DOI:** 10.1155/2021/6619598

**Published:** 2021-03-11

**Authors:** Juan Cao, Wu Zhao, Xin Guo

**Affiliations:** ^1^School of Mechanical Engineering, Sichuan University, Chengdu 610065, China; ^2^Innovation Method and Creative Design Key Laboratory of Sichuan Province, Chengdu 610065, China

## Abstract

Design fixation is related to the broad phenomenon of unconscious cognition bias that hinders the generation of creative solutions during the conceptual design process. While numerous research studies have gone into the study of design fixation, the experimental methods used were external to the cognitive process of designers; thus, there are some limitations. To address these limitations, the present study utilized electroencephalography (EEG) to explore the differences in neural activities between designers with different degrees of design fixation during creative idea generation. *Fluency*, *flexibility*, and *the degree of copying* were used to evaluate the design performance and fixation degrees of all participants; for the follow-up analyses on brain activity patterns, participants were then divided into the Higher Fixation Group and the Lower Fixation Group according to the evaluation of *the degrees of copying*. Next, participants in each group were contrasted separately against the task-related alpha power changes during creative idea generation. The comparison results revealed that participants with lower design fixation demonstrated stronger alpha synchronization in frontal, parietotemporal, and occipital regions during creative idea generation, while participants with higher design fixation showed stronger task-related alpha desynchronization in frontal, centroparietal, and parietotemporal regions. Such findings suggested that participants with higher fixation showed lower solution *flexibility* because of the inability to inhibit the solutions generated overrelying on intuition. These results could contribute to a deeper understanding of design fixation from the neuroscience perspective and provide essential theoretical supports for the subsequent defixation methods and tool development.

## 1. Introduction

In studies on design inspiration and design innovation, some researchers have revealed the design fixation effect on providing example solutions as an external stimulus to designers during creative idea generation [1–3]. Design fixation, which was originally defined as the designer blindly adhered to a set of limited ideas or concepts during conceptual design [4], would restrict designers' creative thinking and hinder the creation of novel solutions [5]. As design studies continue to attract researchers and scholars from a wide range of technical fields, design fixation has been commonly mentioned in studies of creativity and customarily referred to the situation where designers' creative thinking was restricted due to the overreliance on a series of existing design solutions or the knowledge related to present design problems [5–7]; it is unconsciously [3, 8] and happened to both expert and novice designers [3, 9]. Design fixation is thought to occur at the very early stage of the design process, the conceptual design process [3–5], during which solutions generated have a decisive effect on the ultimate outcomes. Simply stated, the occurrence of fixation would impede the conceptual design process, which would thus lead to strong convergence and lower novelty of solutions or concepts generated by designers, while innovation is the core of the conceptual design. To resolve this conflict, it is important to have a better understanding of creativity in design and the barriers that block it [10–12]. Existing design fixation researches have already made good progress. However, the experimental techniques used are quite homogeneous [2] and are external to the internal cognitive process of the participants; thus, there are some limitations in exploring the cognitive activities during the occurrence of design fixation. The present study utilized EEG technology, which could record the neural responses of participants in real-time and noninvasively, aiming to deeply explore the neural activities involved in the occurrence of different degrees of design fixation.

Crilly and Cardoso [11] highly summarized that design fixation is a state where designers had an unconscious bias due to previous experience, knowledge, or assumptions, which leads to the limited exploration of design space when fulfilling a design task. To explain such cognition bias in design researches, the dual-processing model of cognition which shows the existence of two distinct systems (one is the intuition system (System 1) and the other is the rationality system (System 2)) of thinking [13–15] was introduced in the present study. This offered an interesting perspective in design fixation studies: during the creative idea generation process, through System 1, solutions were generated relying on intuition, experience, and memories. Then, System 2 tries to inhibit these intuition-generated solutions through reasoning and calculation or directly following the solutions proposed in System 1. When System 2 directly follows System 1 without any further consideration, design fixation occurs, and this is what researchers called following “the path of least resistance” [16–19]. Therefore, design fixation during creative idea generation occurred because the participants failed to inhibit the solutions generated relying on intuition. This assumption could be confirmed by the experimental results of some typical design fixation studies [20, 21]. And in the present study, we want to further identify this assumption from the perspective of neuroscience.

Nowadays, EEG has become a very powerful and convenient method to investigate cognitive processes, which plays an important role in the creativity of researches. Research studies on the relationships between activities of EEG alpha-band (8–13 Hz) and creative idea generation produced some reliable, consistent, and robust views on the roles of the task-related alpha-band (synchronization or desynchronization) during the creative idea generation [22, 23]. However, there are few studies on the intersection of EEG and design researches, especially on design fixation. Recently, Camarda et al. [24] have gone into the study of the relationships between functional fixedness and alpha-band power changes in both the frontal and temporoparietal regions during creative idea generation using the Alternative Uses Task adapted for EEG recording. However, it should be noted that in the study of Camarda et al. [24] participants were only required to complete the Alternative Uses Task without any additional problem-solving task or the specific design task. This made their findings limited and might not be applicable to fixation effects induced in other contexts such as the design fixation effect. Therefore, in the present study, participants were asked to finish a specific design task, and we wanted to determine whether the findings observed in their study could also be identified in the present study.

The present study aimed to explore neural activity patterns of designers with higher and lower fixation during creative idea generation through EEG technology. Participants were required to generate as many solutions as possible to finish the design task under the same condition. The design fixation degrees and design performance of all participants were rated by evaluating their solutions according to three evaluation metrics: *fluency*, *flexibility*, and *the degree of copying*. Based on the evaluation results, the participants were divided into the High Fixation Group and Low Fixation Group. Then, the task-related alpha-band activity patterns in different brain regions and hemispheres of participants in the two groups and cognitive activities involved in the solution creation were analyzed. Using the real-time recording neuroscientific methodology, it is possible to discover deeper insights into design fixation compared to the typical behavioral methods used in existing design researches. Furthermore, the findings of the present study will provide essential theoretical supports for the subsequent defixation methods and tools development and, meanwhile, will contribute to the further application and interpretation of EEG technology in future studies on related fields.

The rest of the paper is structured as follows. In Section 2, we described the experimental setup, the design task, the participants, and the experiment equipment. Besides, solution evaluation metrics and analyses, the EEG data acquisition and analyses, were also described.  Section 3 presented the comparison results between participants with higher and lower fixation in terms of design performance and task-related alpha-band activity patterns, followed by discussions and limitations on the present study in Section 4. In Section 5, the findings of the present study were summarized.

## 2. Materials and Methods

The main objective of this experiment was to explore the neural activity patterns of participants with different degrees of design fixation while generating creative ideas. According to recent researches in this field, the occurrence of design fixation during creative idea generation might result from the failure to inhibit the solutions generated relying on intuition; therefore, we could generally assume that participants with different degrees (higher vs. lower degree) of design fixation have different brain activity patterns especially alpha-band activity patterns during the creative idea generation process. Details are as follows.

### 2.1. Participants

A total of 23 mechanical engineering postgraduate students at Sichuan University participated in the experiment. Two participants were excluded from further analysis because of the missing data due to the excessive impedance, so the final sample consisted of 21 participants (9 females, aged 22–29 years; *M* = 26.71 years; SD = 1.75 years). They all had at least 5 years of engineering experience. All participants were healthy, were right-handed, had normal vision or vision corrected by wearing glasses or contact lenses, and had no reported history of neurological or psychiatric disorders. They all gave written informed consent for the EEG experiment. The experiment was carried out following the rules of the human research ethics committee of Sichuan University. And all participants received an honorarium for their participation after the experiment.

### 2.2. Design Task

**Figure 1:**
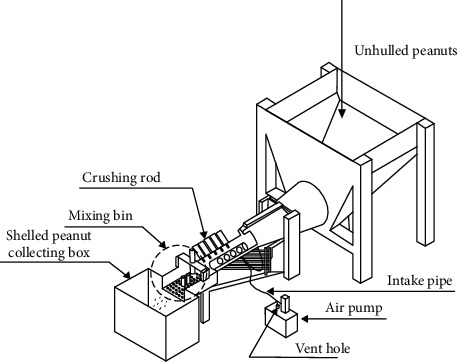
The example solution from the paten document (CN109288079A).

The task is to design a device to shell peanuts, and the device should satisfy some requirements: (1) not destroying peanut kernels, (2) ability to remove a lot of peanut shells, (3) easy to manufacture, and (4) low cost. All participants were provided with the same design requirements and the same example solution as shown in Figure 1, which illustrates one way to finish this design task. The example solution is derived from a Chinese Patent for Invention (Published Application Number: CN109288079A) titled “A peanut shelling device.” The design task was set up for two reasons: the first was that this design task is an engineering design problem that was suitable for all the participants; the second reason was that none of the participants had been exposed to such a design problem before.

### 2.3. The Experiment Equipment and Procedure

BrainProductTM actiChamp-32 Research Amplifier with a sampling rate of 50 kHz and an impedance of 20 kΩ was used to collect EEG data. The EEG signals were recorded by 33 electrodes, and the electrode positions were in line with the international 10-20-system standard.

**Figure 2:**
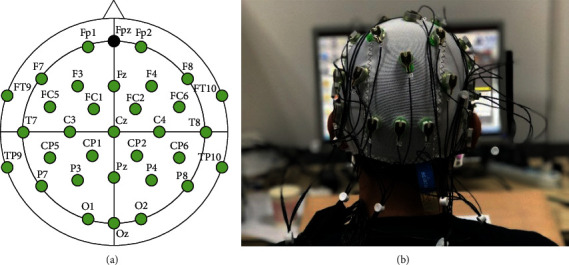
The electrode distributions (a) and a participant finishing the design task (b).

For subsequent statistical analysis of task-related alpha-band activity patterns, electrode positions were aggregated as follows: frontal (F) left (Fp1, FT9, F3, F7) right (Fp2, FT10, F4, F8), frontocentral (FC) left (FC1, FC5) right (FC2, FC6), centrotemporal (CT) left (C3, T7) right (C4, T8), centroparietal (CP) left (CP1, CP5) right (CP2, CP6), parietotemporal (PT) left (P3, P7) right (P4, P8), and occipital (O) left (O1) right (O2). The alpha power of each brain region is the mean power of all electrodes in this brain region. For the analysis of potential hemispheric differences, the midline electrodes (Fz, Cz, Pz, and Oz), ground electrode (Fpz) which was placed on the center of the forehead, and reference electrodes (TP9, TP10) which were, respectively, placed at left and right mastoids were not included in the analysis. The positions of the electrodes are shown in Figure 2.

**Figure 3:**
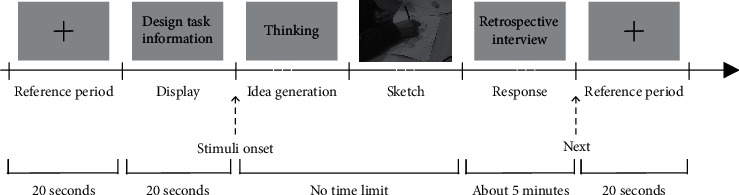
The experiment procedure.

For each participant, a preexperiment was conducted to let them get familiar with the experimental procedure and experiment equipment and, more importantly, find a comfortable position to finish the design task. The whole procedure of the experiment is shown in Figure 3.

Each experiment started with eyes closed for 20 seconds, which aimed to let the participant calm down, and then with eyes open for 20 seconds during which the EEG signals recorded served as the reference to assess brain activities during the idea generation process. Then, the design task follows. Every participant was explicitly informed of their task in detail at the beginning of the design task. When the participant was sure to understand the design task, the example solution was displayed on the screen. Participants observed the example solution and then attempt to come up with their solutions. When they were sure to have their solutions, they could sketch them. They were required to generate as many solutions as possible and write the design instructions for their sketch solutions. As there is no time limit, the participants can end the design task whenever they thought they finished it. Then, a brief, five-minute retrospective interview was conducted. The whole process of the experiment was videotaped.

### 2.4. EEG Data Acquisition and Preprocessing

Throughout the experiment, we mainly recorded and analyzed the EEG data during the idea generation period, and the EEG data during the sketching period were not included. EEG data were recorded through BrainVision Recorder and preprocessed through BrainVision Analyzer2.1 software (BrainProduct Inc.). The power of EEG is reflected by the calculation of the power spectral density (PSD). To eliminate the high-frequency EMG (Electromyography) signals generated by the body movements of participants and EOG (Electrooculogram) signals produced by eye movements such as blinking, the raw EEG data were firstly filtered, and the filtering range is 0.1 to 40 Hz. Then, the Principal Component Analysis (PCA) was conducted to remove the ocular artifacts. Finally, the processed EEG data calculated the PSD using a fast Fourier transform (FFT). The power obtained in the experimental reference interval is the reference power, and the power obtained in the idea generation interval is the activation power. For quantifying task-related power (TRP) changes in EEG alpha power at electrode *i*, the (log-transformed) power during the reference interval (Pow_*i*_ reference) was subtracted from the (log-transformed) power during activation interval (Pow_*i*activation_), according to the following formula [23]: TRP_*i*_ = log (Pow_*i*activation_) − log (Pow_*i*reference_). When the TRF is positive, it means that the power during the activation interval is increased as compared with that during the reference interval, and the power increases are expressed as synchronization. Contrariwise, decreases in power from the reference to the activation interval are expressed as desynchronization. The TRP changes refer to a situation in which brain activities when performing a specific cognitive task (in the present study, the creative idea generation) are associated with the reference interval when performing no task. This can ensure that the differences in TRP are due to the specific task rather than baseline brain differences of individuals' brains.

### 2.5. Metrics

An important difference between design fixation evaluation metrics is whether they are evaluated subjectively (subjective metrics) or objectively (objective metrics). The selection of evaluation metrics mainly depends on the study aims, experiment tasks, and hypotheses. The more subjective metrics are mainly inclined to the judgment of evaluators, while the relatively objective metrics tend to be directly calculated [25]. In the present study, the authors mainly focused on the design outcomes; considering the experimental task conducted, three evaluation metrics *fluency*, *flexibility*, and *the degree of copying* were selected to evaluate all solutions generated per participant. The three metrics all could be explicitly measured, primarily to quantitatively evaluate the design fixation and design performance of each participant: (1) *fluency*, a metric to evaluated participants' design performance, was measured by counting the total number of ideas generated per participant, (2) *flexibility*, which was also a metric to evaluated participants' design performance, was calculated by counting the number of idea categories generated per participant, (3) *the degree of copying*, a metric measured design fixation, was evaluated by calculating the percentage of features from example solutions participants used in their design solutions. In addition, the *time* the participants spent generating ideas was recorded as an additional metric to evaluate participants' design performance. The two independent evaluators evaluated all solutions generated per participant according to the features listed in Table 1. Considering the design task in this study, and following the study of Linsey et al. [3], the basic features of the example solution in this study were categorized as shown in Table 1.

For subsequent analyses, participants would be classified into two groups based on the median value of *the degree of copying*. Those with scores higher than the median value of *the degree of copying* would be classified as the High Fixation Group (*G*_HF_), while those with scores lower than the median would be classified as the Low Fixation Group (*G*_LF_). It should be noted that using the median of evaluation scores on creativity outputs was commonly used to divided participants into two groups in related studies [22, 24, 26]. After grouping, separate analyses of the High Fixation Group (*G*_HF_) and Low Fixation Group (*G*_LF_) were performed according to each metric.

### 2.6. Statistical Analysis

#### 2.6.1. Statistical Analysis of Metrics

The two participants were excluded from further analysis, resulting in the final sample that consists of 21 participants. And two independent evaluators rated the solution sketches generated per participant. To ensure the reliability of evaluations for all metrics, an interevaluator agreement was performed by two independent evaluators, and Pearson's correlation was used to determine the interevaluator reliability. And to evaluate the design performance of participants with different fixation degrees, the one-sample Kolmogorov-Smirnov test was first used to verify the normality of the evaluation results of the evaluation metrics. Secondly, Levene's test was used to check the homogeneity of variance. When the data were normally distributed and the variance was homogeneous, the ANOVA (Analysis of Variance) would be carried out. When the data did not match the normal distribution, a Nonparametric Kruskal-Wallis Test was performed to get further analysis. For the analysis of the time the participants with different fixation degrees spent generating ideas, an independent sample *T*-test was conducted. The *P* value of significance level was 0.05, which was the default value of the system. All statistical analyses of solutions were conducted in IBM SPSS statistics package version 24 for Windows. And the results of the evaluators' agreement on each metric were reported, respectively, hereinafter.

#### 2.6.2. Statistical Analysis of Alpha-Band Activity Patterns

The task-related alpha power changes of participants in the two groups (*G*_LF_ vs. *G*_HF_) were analyzed. Specifically, the brain activation regions of participants with different fixation degrees were first compared. Then, the homogeneity tests were conducted, the sample data obeyed multivariate normal distribution, and, therefore, the repeated measures multivariate analysis of variance (MANOVA) was conducted to explore the differences of activity patterns of alpha-band TRP changes (synchronization or desynchronization), respectively, considering HEMISPHERE (Left vs. Right) and REGION (F: frontal, FC: frontocentral, CT: centrotemporal, CP: centroparietal, PT: parietotemporal, O: occipital) as within-subject factors and FIXATION DEGREE (*G*_LF_ vs. *G*_HF_) as a between-subject factor. For the repeated measures MANVOA, to prevent the interference of the sphericity assumption, the Greenhouse-Geisser method was performed to correct the degree of freedom. The *P* value of significance level was 0.05, which was the default value of the system, and the partial eta squared (*ηp*^2^) was used to access the effect size. All the statistical analyses of alpha-band activity patterns were conducted using IBM SPSS statistics package v24 for Windows.

## 3. Results

### 3.1. Results of Metrics

Separate evaluations of all solutions generated by the 21 participants were performed for each of the metrics described in the previous section. The higher Pearson's correlation indicated that the two evaluators gave highly similar scores. Pearson's correlation coefficient between the two evaluators on *fluency* was significantly high (*R* = 0.97, *P* < 0.01), which showed that the measurement of *fluency* was highly reliable. Also, a strong correlation (Pearson's correlation coefficient, *R* = 0.77, *P* < 0.01) was observed between the two evaluators on the *flexibility*, which showed that the measurement for this metric was reliable. For *the degree of copying*, Pearson's correlation coefficient is 0.96 (*P* < 0.01), so the measurement of this metric was reliable.  Table 2 shows the evaluation results of solutions generated by all participants.

The analysis of the one-sample Kolmogorov-Smirnov test on design fixation degrees showed that the design fixation degrees of participants in this study accorded with a normal distribution (*N* = 21, *M* = 0.41, SD = 0.20, *Z* = 0.14, *P* > 0.05). Therefore, for the subsequent analyses of EEG signals of participants with different fixation degrees during creative idea generation, we select the median value (0.41) of *the degree of copying* to group participants into the Low Fixation Group (*N* = 12, *M* = 0.26) and the High Fixation Group (*N* = 9, *M* = 0.60). After grouping, separate analyses of the High Fixation Group (*G*_HF_) and Low Fixation Group (*G*_LF_) were performed according to each evaluation metric, and the results were shown in Table 3. The initial results showed that participants in G_HF_ generated a lower number of ideas and idea categories, reused a higher number of features from the example solution, and spent less time generating ideas compared to participants in G_LF_.

Further analyses on the *fluency* and *flexibility* of ideas generated by participants with different fixation degrees (*G*_LF_ vs. *G*_HF_) were also conducted to evaluate participants' design performance. The variance of *fluency* data was not homogeneous, so a Kruskal-Wallis ANOVA was conducted, and the results showed that there was no significant correlation between the fixation degrees (*G*_LF_ vs. *G*_HF_) and *fluency* (*H* = 0.16, DF = 1, *P* > 0.05, *N* = 21), although participants in G_LF_ generated a few more solutions than those in *G*_HF_ (*G*_LF_: RM = 11.42; *G*_HF_: RM = 10.44). Besides, the variance of *flexibility* data was not homogeneous either, so another Kruskal-Wallis ANOVA was implemented. The results revealed high correlations of high significance between the fixation degrees (*G*_LF_ vs. *G*_HF_) and *flexibility* (*H* = 11.07, DF = 1, *P* > 0.05, *N* = 21), and participants in *G*_LF_ (RM = 14.63) demonstrated higher levels of solution *flexibility* compared with participants in *G*_HF_ (RM = 6.17). The results of the independent sample *T*-test on the *time* data showed no significant difference in idea generation time between participants in *G*_LF_ and *G*_HF_ (T (19) = 0.63, *P* > 0.05, *D* = 0.28).

### 3.2. Results of Alpha-Band Activity Patterns

**Figure 4:**
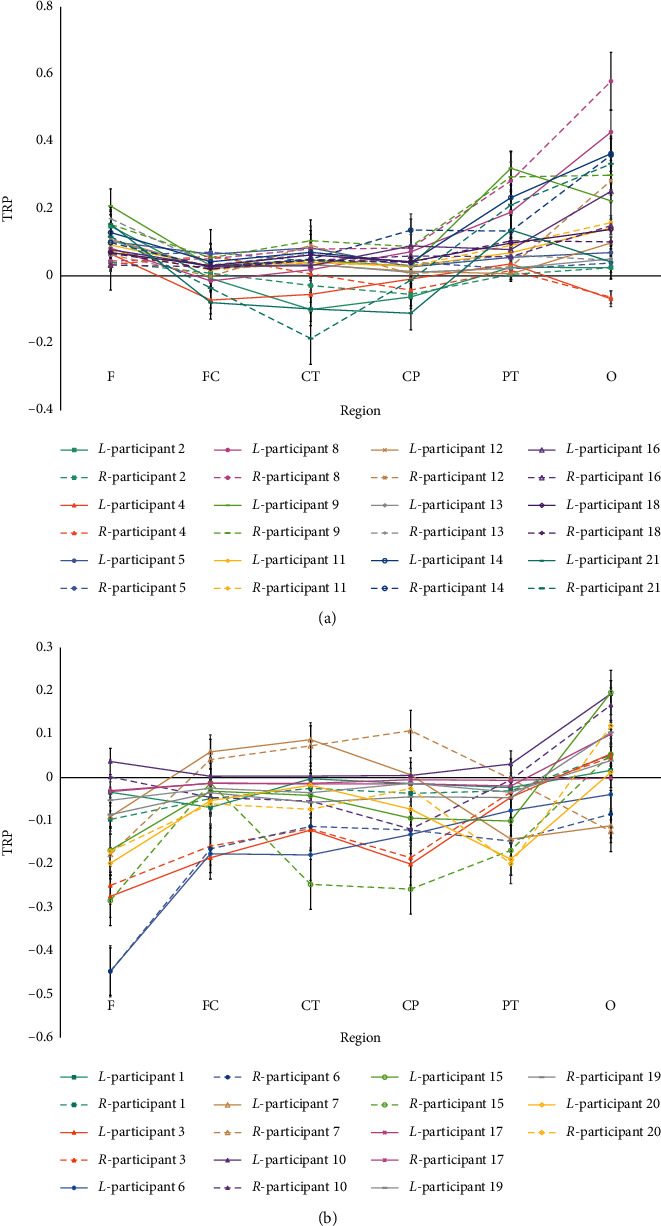
The alpha-band TRP changes in different hemispheres and regions of all participants in *G*_LF_ (Above, *N* = 12) and *G*_HF_ (Below, *N* = 9).

**Figure 5:**
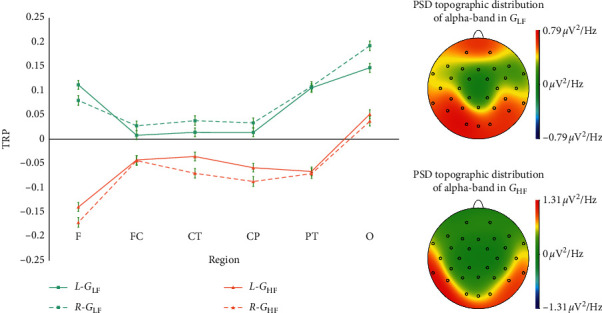
The PSD topographic distribution and alpha-band TRP changes after the average processing of sample data.

Individually, participants in each group (*G*_LF_ vs. *G*_HF_) were contrasted separately against brain activation during creative idea generation.  Figure 4 showed alpha-band task-related power (TRP) changes in the different hemispheres (*L* = left hemisphere, *R* = right hemisphere) and different regions (F: frontal, FC: frontocentral, CT: centrotemporal, CP: centroparietal, PT: parietotemporal, O: occipital) of all participants in *G*_LF_ (*N* = 12) and *G*_HF_ (*N* = 9) during creative idea generation. The average processed TRP changes and the PSD topographic distribution of alpha-band in different hemispheres and regions of participants in *G*_LF_ (*N* = 12) and *G*_HF_ (*N* = 9) were illustrated in Figure 5.

These data revealed that the alpha-band mainly activated in F (frontal), PT (parietotemporal), and O (occipital) regions of participants in *G*_LF_ and was more active in the left hemisphere than in the right one. It was also revealed that the alpha-band mainly activated in PT (parietotemporal) and O (occipital) regions of participants in *G*_HF_ and showed a more active state in the right hemisphere than in the left one. Besides, participants in *G*_LF_ maintained larger alpha-band synchronization, while participants in *G*_HF_ maintained larger alpha-band desynchronization. To explore alpha-band activity patterns in more detail, further statistical analyses were performed.

The repeated measures MANOVA revealed some significant results on the differences of activity patterns of alpha-band TRP. (i) A significant main effect of REGION (*F* (1, 19) = 14.484, *P* < 0.01, partial‐*η*^2^ = 0.433) on alpha-band TRP changes was revealed, reflected in the larger task-related alpha-band synchronization in the PT (parietotemporal) region and O (occipital) region. (ii) There was no significant main effect of HEMISPHERE (*F* (1, 19) = 0.138, *P* > 0.05, *ηp*^2^ = 0.007) on alpha-band TRP changes. (iii) A significant main effect of FIXATION DEGREE (*F* (1, 19) = 26.745, *P* < 0.01, *ηp*^2^ = 0.585) on alpha-band TRP changes was revealed, with the larger alpha-band synchronization of participants in G_LF_ and the larger alpha-band desynchronization of participants in G_HF_. (iv) There were significant interactions between FIXATION DEGREE × REGION (*F*_FIXATION DEGREE × REGION_ (1, 19) = 7.207, *P* < 0.05, *ηp*^2^ = 0.277) and REGION × HEMISPHERE (*F*_REGION × HEMISPHERE_ (1, 19) = 4.938, *P* < 0.05, *ηp*^2^ = 0.206), embodied in the larger task-related alpha-band synchronization in the F (frontal), PT (parietotemporal), and O (occipital) regions of participants in *G*_LF_. In the F (frontal) regions, the task-related alpha-band synchronization of the left hemisphere was stronger than that of the right hemisphere. In other brain regions, the opposite was shown; that is, the task-related alpha-band synchronization was stronger in the right hemisphere over these brain regions than in the left one. In *G*_HF_, the larger task-related alpha-band desynchronization was stronger in the right hemisphere over F (frontal), CP (centroparietal), and PT (parietotemporal) regions of participants in G_HF_. However, no significant interaction between FIXATION DEGREE and HEMISPHERE (*F*FIXATION DEGREE × HEMISPHERE (1, 19) = 4.093, *P* > 0.05, *ηp*^2^ = 0.177) on alpha-band TRP changes was found. (v) Significant interactions between REGION × HEMISPHERE × FIXATION DEGREE ((_*F*REGION × HEMISPHERE × FIXATION DEGREE_ (5, 95) = 4.490, *P* < 0.05, *ηp*^2^ = 0.191) were found on alpha-band TRP changes. (vi) The interactions of REGION × HEMISPHERE were significant on TRP changes of participants in *G*_LF_ (*F* (1, 11) = 6.922, *P* < 0.05, *ηp*^2^ = 0.386), but no further significant interactions of REGION × HEMISPHERE were found on TRP changes of participants in *G*_HF_ (*F* (1, 8) = 0.258, *P* > 0.05, *ηp*^2^ = 0.031). And in *G*_LF_, i larger alpha synchronization was found in the right hemisphere than in the left (Left: *M* = 0.067, SE = 0.016; Right: *M* = 0.080, SE = 0.017).

## 4. Discussion

The present study aimed to explore the neural activities involved in different degrees of design fixation during creative idea generation. Given the consistent findings of neuroscience studies on creativity, we focused on task-related power changes in the alpha-band. Therefore, an EEG experiment was conducted to explore the task-related alpha-band activity patterns in different hemispheres and regions of participants with high and low fixation degrees during creative idea generation. The participants were required to generate as many solutions as possible to finish the design task and they were provided with the same example solution. *Fluency*, *flexibility,* and *degree of copying* were used to evaluate the design performance and fixation degrees of solutions generated per participant. Besides, the time participants spent generating ideas was also analyzed to evaluate participants' design performance. And the alpha-band activities of every participant during idea generation were recorded and analyzed.

The solution evaluation results revealed no significant relationship between the fixation degrees and *fluency*. In general, an increase in idea fluency is usually considered positive [27], and some studies regarded the decrease of fluency as an indicator of fixation; usually the lower the fixation degree, the higher the level of solution fluency [25, 28, 29]. Nonetheless, in the present study, no significant correlations between *fluency* and the fixation degrees were found, and this might indicate that the lower level of *fluency* might not be a typical characteristic of design fixation. At the same time, no significant difference was found in time spent generating ideas between participants with different fixation degrees; this is consistent with the results from the study of Neroni and Crilly [30]. One possible explanation is that during the whole process of idea generation, different participants have different focuses and design patterns. Further researches concerning the design process and design fixation are needed to verify this argument.

Critically, we observed a high correlation of great significance between idea *flexibility* and design fixation degree. This finding was consistent with most design fixation studies where design fixation was mainly manifested in the decrease of solution categories [1–4, 6, 9, 10]. *Flexibility* reflected the ability to switch between diverse fields to explore alternative solutions. That the participants in G_HF_ demonstrated lower *flexibility* could be well understood because they conducted a limited exploration of solution space due to the overreliance on the given example solution without consideration of other alternative solutions. Given the important role of inhibitory control in the generation of creative ideas [19, 24, 31, 32], this result can be further explained as follows: participants with higher degrees of design fixation unconsciously followed the path of least resistance during creative idea generation, reflected in the inhibitory control function of System 2 failed to work; as a result, more solutions with higher degrees of copying generated intuitively in System 1 were revealed. Of course, following the path of least resistance, and generating solutions by searching for the given, established solutions would be far easier and required less cognitive effort [33, 34]. Participants with lower degrees of design fixation were able to stray far from the path of least resistance, and the inhibitory control function of System 2 worked to inhibit the intuitive, common, and higher-level copying solutions generated in System 1. Critically, these claims can be proved by electrophysiological results in the present study.

Consistent with the findings of previous neural findings [19, 24, 35], the electrophysiological results in the present study revealed that task-related alpha-band activity patterns were significantly different in participants with different degrees of design fixation during creative idea generation. We found that participants with lower fixation degrees demonstrated task-related alpha synchronization in frontal, parietotemporal, and occipital regions, and critically, the right hemisphere showed larger alpha-band synchronization during ideation. While participants with higher degrees of design fixation showed stronger task-related alpha desynchronization in frontal, centroparietal, and parietotemporal regions. Specifically, the right hemisphere displayed larger alpha-band desynchronization. Given that the frontal alpha synchronization reflects the function of inhibitory control of task-irrelevant areas [36] and the obvious ideas generated relying on intuition [26, 37], our results provided additional support for the role of the inhibitory control in creative idea generation. Besides, the parietal and occipital alpha synchronizations reflect the shielding mechanism that prevents interference from unrelated external stimulus and supports internally directed attention [23, 32, 38]; our results further revealed that, in addition to the inhibitory control, the participant with a lower fixation degree also maintained a high degree of internally directed attention.

To sum up, our results confirmed that design fixation occurrence was closely related to the inability to inhibit the solutions generated relying on intuition during creative idea generation and proved that inhibitory control was the core process of idea generation to generate creative solutions. Besides, it should be noted that alpha synchronization is also closely associated with divergent thinking [23, 26, 37, 39] which usually refers to the thinking process of generating original ideas by exploring various possible solutions. Consequently, our results could further explain why the participant with a higher fixation degree demonstrated lower solution flexibility because the higher fixation restricted their divergent thinking. Therefore, the occurrence of high-level design fixation may be more reflected in the flexibility rather than fluency of the generated solutions. Note, however, that generating a creative idea is a complex activity that covers many cognitive activities. Therefore, we assume that the generation of creative ideas involving the inhibitory control on the fixation effect may not simply require activities in certain brain regions or a hemisphere but also requires the activities of networks of multiple regions of the brain. Future studies in this field are thus particularly challenged to explore how the networks of multiple regions affect the generation of creative ideas involving inhibitory control on the fixation effect.

Nevertheless, the current study also has some limitations that should be acknowledged. Firstly, it should be acknowledged that the ideal setup of this experiment is to add a control group where participants would be provided with no example solution; this is thought to be more scientific. However, in the very early stage of our research, several experiments were carried out without an example solution, the results showed that most of the participants were at a loss facing such a design task without any hints. They exhausted their effort to comprehend the design task, but finally, almost none of them generated any ideas. Therefore, we removed the control group where no example solution was provided to participants, but this might affect the comparison results, future studies in this field need to set up a control group, which would be more scientific. Secondly, the EEG signals during sketching were not included in the present study. However, there would be considerable differences in alpha-band activity patterns of participants with different fixation degrees between the idea generation period and sketching period. We will explore this assumption in our future work related to the design process. Thirdly, the results obtained in the present study were under laboratory conditions; in real-world design activities, different findings might be observed. Therefore, further studies are needed to verify whether the findings observed in the laboratory can be generalized to real-world design activities. Finally, the differences in the experience and the knowledge level of participants in our study were not carefully considered; however, both would affect the experiment results. These factors should be considered in future researches to address the limitations of the present study.

## 5. Conclusions

The present study utilized EEG to explore neural activity patterns of designers with higher and lower fixation during creative idea generation and aimed to find some neural basics of design fixation. Given the consistent findings of neuroscience studies on creativity, the present study especially focused on task-related alpha-band power changes of participants with different degrees of design fixation during creative idea generation. The electrophysiological results showed significant differences in participants with higher and lower fixation and proved that the occurrence of high-level design fixation, when faced with an example solution, reflected the inability to inhibit the solutions generated overrelying on intuition. Besides, the observed task-related changes of alpha power in the process of ideation revealed that design fixation may also associate with the defocused internal attention and the restricted divergent thinking. These results could contribute to a deeper understanding of design fixation from the neuroscience perspective, revealing the different neural activities involved in the occurrence of higher and lower degrees of design fixation. Moreover, the findings provide essential theoretical supports for the subsequent defixation methods and tool development and, meanwhile, will contribute to the further application and interpretation of EEG technology in future studies on related fields.

## Figures and Tables

**Table 1:** The basic features of the example solution.

Function	Features from example solution
(Material)	
Guide	Sloped surface
	Conveyor
Import	Hopper
Remove (shell)	Crushing rod
Separate (nut and broken shell)	Winnowing
Store	Bin/basket
Position	Table legs
(Energy)	
Convert	Mechanical energy

**Table 2:** The evaluation results of solutions generated by all participants (*N* = 21).

Metric	Mean (SD)	Std err.	95% LB	95% UB
Fluency	1.90 (1.22)	0.26	1.43	2.48
Flexibility	0.95 (0.86)	0.19	0.63	1.33
Degree of copying	0.41 (0.20)	0.04	0.33	0.50

**Table 3:** The results of solution evaluation and idea generation time of participants in *G*_LF_ (*N* = 12) and *G*_HF_ (*N* = 9).

	Fluency		Flexibility		Degree of copying		Time (s)	
	*M*	SD	*M*	SD	*M*	SD	*M*	SD
*G*_LF_	1.83	0.80	1.50	0.65	0.26	0.08	193.67	83.91
*G*_HF_	1.56	0.68	0.33	0.47	0.61	0.11	171.00	79.22

## Data Availability

The data used to support the findings of this study are included within the article.
